# The characteristics and relevant factors of Pap smear test use for women with intellectual disabilities in Taiwan

**DOI:** 10.1186/1472-6963-14-240

**Published:** 2014-06-02

**Authors:** Suh-May Yen, Pei-Tseng Kung, Wen-Chen Tsai

**Affiliations:** 1 Department of Health Services Administration, China Medical University, No. 91 Hsueh-Shih Road, Taichung, Taiwan 40402, Republic of China; 2 Department of Chinese Medicine, Nantou Hospital, Nantou, Taiwan, Republic of China; 3 Department of Healthcare Administration, Asia University, Taichung, Taiwan, Republic of China

**Keywords:** Intellectual disabilities, Disability, Preventive health services, Pap smear test

## Abstract

**Background:**

This study examines the Pap smear usage conditions and relevant influential factors for 18,204 women aged 30 years and above with intellectual disabilities, using nationwide data from 2008.

**Methods:**

The research method of this study is secondary data analysis. The data was obtained from three nationwide databases from 2006 to 2008. This study employed descriptive statistics to analyze the use and rate of Pap smear testing by women with intellectual disabilities. Chi-square test was used to assess the correlation between Pap smear test usage and several variables. Logistic regression analysis was employed to explore the factors that influence Pap smear test usage.

**Results:**

The results show that 4.83% (n =880) of women with intellectual disabilities underwent Pap smear tests. Pap smear test usage rates exhibit a declining trend with increases in age. Factors that significantly influence Pap smear test use include age, urbanization level of resident area, monthly salary, aboriginal status, marital status, existence of DM, severity of disability.

**Conclusions:**

The women with intellectual disabilities had a low use rate of Pap smear test, which is significantly less than the 28.8% usage rate for the general population of women aged 30 years and above.

## Background

Cervical cancer is the most prevalent type of cancer occurring in women worldwide. The 2009 standardized incidence ratio (SIR) for cervical cancer in women of Taiwan was 11.87 per 100,000 people (n = 1,796) [1]. Regular Pap smear test screening to detect early stage cervical cancer is proven to effectively reduce the mortality rate of cervical cancer [2]. Studies have shown that conducting a Pap smear test once every three years can reduce the incidence and mortality rates of cervical cancer by 60% to 90% [3]. A study conducted in Hong Kong (2007) indicated that with consistent screening (once every three years) and 100% coverage, the number of disability-adjusted life years per year is reduced by 594 [4]. Since 1995, Taiwan’s National Health Insurance (NHI) has offered one free Pap smear test per year to women aged 30 and above, and if anomalies are found, follow-up diagnosis and treatment is arranged to prevent the development of cancer. Following the promotion of this policy, the mortality rate for cervical cancer in 2010 (standardized mortality rate of 4.4 per 100,000 people) has declined two-fold from that in 1996 (standardized mortality rate of 10.5 per 100,000 people) [5].

Previous studies have demonstrated that compared to the general population of women, those with disabilities are less likely to receive regular cervical cancer screening [6-8]. A 2008 study conducted by the U.S. Behavioral Risk Factor Surveillance System indicated that the Pap smear test usage of women with disabilities (78.9%) was significantly lower than that of the general population of women (83.4%) [9]. In a U.K. study, Reynolds et al. [10] proposed that women with intellectual disabilities were significantly more likely to be neglected by screening programs. Pearson et al. [11] investigated Pap smear test usage in Exeter (England southwestern city) and found that for five years only a quarter of eligible women with learning disabilities underwent cervical screening. This differs substantially from that for the general population of women (82%) [11]. Stein and Allen [12] obtained similar results, reporting that only 13% of women with learning disabilities who were eligible for the Pap smear test underwent at least one Pap smear test. By contrast, the rate for the general population of women was 88% [12].

In late 2011, 98,046 people in Taiwan were reported to exhibit intellectual disabilities, accounting for 0.4% of the total population [13]. Regarding people with intellectual disabilities, 89.5% live with family members [14]. As specified in People with Disabilities Rights Protection Act, the Taiwanese government provides special education, social welfare, and health care supports for intellectually disabled person. People with an IQ (intelligence quotient) score of below 70 who are evaluated as being intellectually challenged by government-recognized physicians and psychologists are issued a disability card.

People with intellectual disabilities experience substantially more obstacles in health promotion and cancer prevention activities compared to the general population. This assertion was described in a study conducted in Australia, which explored general practitioners’ perceptions regarding the obstacles to health care experienced by people with intellectual disabilities and the relevant solutions. The results indicated that the obstacles included communication difficulties between patients and paramedics, patients’ poor compliance with management plans, inspection difficulties, and an insufficient understanding of available resources [15].

In a previous study, we investigated the Pap smear test used among women with any type of disability aged over 30 years and observed that women with intellectual disabilities exhibited the lowest use rate (4.83%) [16]. Thus, the objective of this study was to examine the use of Pap smear tests among women with intellectual disabilities in Taiwan, and the relevant influential factors.

## Methods

### Data source and participants

This study referenced 2008 data from the Ministry of the Interior and adopted a study population of 18,204 women aged 30 years and above, who had intellectual disabilities and possessed a disability certificate [17]. The criteria used to define intellectual disabilities in Taiwan are based on the definition of the American Association on Mental Retardation [18].

Data was obtained from the following three sources: (a) Pap smear test data (2008) were obtained from the Health Promotion Administration, Ministry of Health and Welfare, Taiwan; (b) medical claims data from the National Health Insurance Research Database were provided by the Ministry of Health and Welfare, Taiwan; and (c) information of disabled individuals from the 2008 Registry of Disabled People were obtained from the Ministry of the Interior. All three data sets were compiled and managed by the Statistics Center of the Ministry of Health and Welfare, Taiwan. We performed all analyses at the Statistics Center and were unaware of the identities of the people from whom the data were collected. The Institutional Review Board of China Medical University and Hospital approved this study (IRB No. CMU-REC-101-012).

### Variables description

The relevant variables in this study include (a) demographic variables: age, education level, marital status, premium-based monthly salary, and aboriginal status; (b) environmental variable: urbanization level of resident area; Urbanization of areas was categorized into eight levels. The Level 1 was the area with the highest level of urbanization, whereas the Level 8 was the areas with the lowest level of urbanization; (c) health status: catastrophic illness/injury, relevant chronic illnesses (including cancer and diabetes); (d) severity of disability: mild (IQ 2 ~ 3 standard deviations below the mean)), moderate (IQ 3 ~ 4 standard deviations below the mean), severe (IQ 4 ~ 5 standard deviations below the mean), and very severe (IQ 5 standard deviations below the mean; (e) use of Pap smear test.

In this study, people with diabetes were defined as those who received a primary or secondary diagnosis according to ICD-9-CM code 250.xx and underwent at least three outpatient visits or one hospitalized treatment for diabetes within 365 consecutive days. We analyzed data collected between 2006 and 2008 to define diabetes. Cancer patients were identified in the NHI catastrophic illness/injury registry file, which contains information on people with any catastrophic illness or injury confirmed by the Bureau of National Health Insurance.

### Statistical analysis

A statistics software package (SAS 9.2) was used for data analysis. Descriptive statistics was first conducted to determine the frequency of Pap smear testing and the variable percentage. A chi-square test was used to assess the correlation between Pap smear test usage and several variables. Finally, logistic regression analysis was employed to explore the factors that influence Pap smear test usage. In this study, a *p* value of less than 0.05 was considered statistically significant.

## Results

### Basic participant characteristics

A total of 18,204 women with intellectual disabilities aged 30 years and above were eligible to undergo free Pap smear testing. The majority of these women were aged between 30 and 49 years (70.21%, n =12,782), and resided in areas with Level 2 of urbanization (18.36%). Additionally, the population comprising participants who possessed premium-based monthly salary of < NT$15,840 (New Taiwan Dollar, NT$) was the highest (47.25%), followed by the dependent population (e.g., parents, children, or spouse) (33.04%). Few participants were of aboriginal ethnicity (1.49%). Their overall education level was low, typically elementary school or lower (61.29%, n =11,158). Regarding their marital status, most of the participants were unmarried (35.90%), followed by those who were married (33.11%). A total of 12.37% of the participants had experienced a catastrophic illness or injuries. For relevant diseases, 1.24% and 8.58% of the participants had cancer and diabetes mellitus (DM), respectively. Regarding the disability severity, the greatest proportion of the participants had moderate disabilities (36.74%), and the number of severely disabled people comprised the smallest proportion (17.52%) (Table 1).

**Table 1:** Use of Pap smear among women with intellectual disability: basic characteristics and bivariate analysis

			**Used**		**Did not use**		**χ**^**2**^
**Variable name**	**N = 18,204**	**%**	**n**_**1**_ **= 880**	**%**	**n**_**2**_ **= 17,324**	**%**	**p-value**
Gender							-
Female	18,204	100.00	880	4.83	17,324	95.17	
Age							<0.001*
30-39 years	7,178	39.43	276	3.85	6,902	96.15	
40-49 years	5,604	30.78	312	5.57	5,292	94.43	
50-59 years	3,491	19.18	209	5.99	3,282	94.01	
60-69 years	1,346	7.39	73	5.42	1,273	94.58	
≧70 years	585	3.21	10	1.71	575	98.29	
Level of urbanization^a^							<0.001*
Level 1	1,684	9.25	55	3.27	1,629	96.73	
Level 2	3,343	18.36	130	3.89	3,213	96.11	
Level 3	2,610	14.34	127	4.87	2,483	95.13	
Level 4	1,692	9.29	73	4.31	1,619	95.69	
Level 5	3,044	16.72	182	5.98	2,862	94.02	
Level 6	2,279	12.52	112	4.91	2,167	95.09	
Level 7	2,425	13.32	126	5.20	2,299	94.80	
Level 8	1,127	6.19	75	6.65	1,052	93.35	
Premium-based monthly salary							0.003*
Dependent population	6,014	33.04	285	4.74	5,729	95.26	
<15,840	8,601	47.25	378	4.39	8,223	95.61	
16,500-22,800	3,287	18.06	193	5.87	3,094	94.13	
24,000-28,800	174	0.96	15	8.62	159	91.38	
30,300-36,300	92	0.51	6	6.52	86	93.48	
38,200-45,800	36	0.20	3	8.33	33	91.67	
Aboriginal							0.011*
Yes	271	1.49	22	8.12	249	91.88	
No	17,933	98.51	858	4.78	17,075	95.22	
Education level							0.819
Elementary school and under	11,158	61.29	546	4.89	10,612	95.11	
Junior high school	3,550	19.50	172	4.85	3,378	95.15	
Senior high/vocational school	1,217	6.69	50	4.11	1,167	95.89	
Junior college/university and above	114	0.63	6	5.26	108	94.74	
Unclear	2,165	11.89	106	4.90	2,059	95.10	
Marital status							<0.001*
Married	6,028	33.11	463	7.68	5,565	92.32	
Unmarried	6,536	35.90	133	2.03	6,403	97.97	
Divorced and widowed	652	3.58	47	7.21	605	92.79	
Unclear	4,988	27.40	237	4.75	4,751	95.25	
Catastrophic illness/injury							0.989
Yes	2,252	12.37	109	4.84	2,143	95.16	
No	15,952	87.63	771	4.83	15,181	95.17	
Relevant diseases							
Cancer							0.004*
Yes	225	1.24	20	8.89	205	91.11	
No	17,979	98.76	860	4.78	17,119	95.22	
Diabetes mellitus							<0.001*
Yes	1,562	8.58	114	7.30	1,448	92.70	
No	16,642	91.42	766	4.60	15,876	95.40	
Level of severity of intellectual disability							<0.001*
Mild	3,461	19.01	274	7.92	3,187	92.08	
Moderate	6,689	36.74	380	5.68	6,309	94.32	
Severe	4,865	26.72	139	2.86	4,726	97.14	
Very severe	3,189	17.52	87	2.73	3,102	97.27	

^a^Level one: the most urbanized areas.

*p < 0.05.

### Pap smear test usage among women with intellectual disabilities

In 2008, only 4.83% (n =880) of women with intellectual disabilities had previously undergone Pap smear tests, which is significantly lower than that (28.8%) for the general population of women aged 30 years or above in Taiwan [19]. The Pap smear test usage rate was the highest among the 50 to 59 years age group (5.99%), followed by the 40 to 49 years age group (5.57%) (P <0.05). The use rate of the Pap smear test was highest for those living in areas with Level 8 of urbanization (6.65%), whereas the lowest use rate was for those residing in areas with Level 1 of urbanization (i.e., the most urbanized) (3.27%) (P <0.05). People with premium-based monthly salary of NT$24,000 to NT$28,800 exhibited the highest Pap smear test usage rate (8.62%), followed by those with premium-based salary of NT$38,200 to NT$45,800 (8.33%) and < NT$15,840 (4.39%) (P <0.05). The usage rate among the aboriginal population (8.12%) was significantly greater than that for the non-aboriginal population (4.78%) (P <0.05).

For marital status, women who were married (7.68%), divorced or widowed (7.21%) exhibited a higher Pap smear test usage rate compared to unmarried women (2.03%). Regarding relevant diseases, the usage rate for women diagnosed with cancer was 8.89%, greater than that for the non-cancer population (4.78%) (P <0.05). Similarly, the usage rate for DM patients (7.30%) also exceeded that for non-DM patients (4.60%) (P <0.05). For the severity of disability, people with severe disabilities (2.73%) exhibited the lowest Pap smear test usage rate, whereas those with mild disabilities (7.92%) showed the highest usage rate. In addition, a negative correlation existed between the severity of intellectual disability and the Pap smear test usage rate (P <0.05) (Table 1).

### Factors that influence Pap smear test usage for women with intellectual disabilities

Table 2 shows the factors that influence Pap smear test use, as estimated using a logistic regression model. The results indicate that the variables significantly affecting Pap smear test use (P <0.05) included age, urbanization level of resident area, premium-based monthly salary, aboriginal status, marital status, existence of DM, and severity of disability.

**Table 2:** Logistic regression models for Pap smear use among women with intellectual disability

	**Unadjusted**				**Adjusted**			
**Variable name**	**OR**	**95% CI**		**p-value**	**OR**	**95% CI**		**p-value**
Age								
30-39 years	-	-	-	-	-	-	-	-
40-49 years	1.47	1.25	1.74	<0.001*	1.19	0.99	1.42	0.058
50-59 years	1.59	1.32	1.92	<0.001*	1.18	0.96	1.46	0.115
60-69 years	1.43	1.10	1.87	0.008*	1.07	0.80	1.43	0.636
≧70 years	0.44	0.23	0.82	0.010*	0.36	0.19	0.69	0.002*
Level of urbanization^a^								
Level 1	-	-	-	-	-	-	-	-
Level 2	1.20	0.87	1.65	0.269	1.00	0.72	1.39	0.987
Level 3	1.52	1.10	2.09	0.012*	1.17	0.84	1.63	0.353
Level 4	1.34	0.94	1.91	0.112	1.05	0.73	1.52	0.782
Level 5	1.88	1.39	2.56	<0.001*	1.42	1.03	1.94	0.032*
Level 6	1.53	1.10	2.13	0.011*	1.15	0.82	1.61	0.428
Level 7	1.62	1.18	2.24	0.003*	1.19	0.85	1.66	0.310
Level 8	2.11	1.48	3.02	<0.001*	1.52	1.04	2.20	0.030*
Premium-based monthly salary								
<15,840	-	-	-	-	-	-	-	-
Dependent population	1.08	0.93	1.27	0.325	1.14	0.95	1.37	0.167
16,500-22,800	1.36	1.14	1.62	0.001*	1.18	0.96	1.45	0.122
24,000-28,800	2.05	1.20	3.52	0.009*	1.79	1.02	3.12	0.041*
30,300-36,300	1.52	0.66	3.50	0.327	1.43	0.61	3.35	0.417
38,200-45,800	1.98	0.60	6.48	0.260	1.77	0.53	5.93	0.352
Aborigine								
No	-	-	-	-	-	-	-	-
Yes	1.76	1.13	2.73	0.012*	1.73	1.08	2.76	0.022*
Educational level								
Elementary school and under	-	-	-	-	-	-	-	-
Junior high school	0.99	0.83	1.18	0.907	1.06	0.87	1.29	0.558
Senior high/vocational school	0.83	0.62	1.12	0.225	1.03	0.74	1.42	0.875
Junior college/university and above	1.08	0.47	2.47	0.856	1.42	0.61	3.31	0.414
Unclear	1.00	0.81	1.24	0.996	0.95	0.76	1.19	0.677
Marital status								
Unmarried	-	-	-	-	-	-	-	-
Married	4.01	3.29	4.87	<0.001*	3.21	2.59	3.97	<0.001*
Divorced or widowed	3.74	2.65	5.27	<0.001*	3.09	2.16	4.42	<0.001*
Unclear	2.40	1.94	2.98	<0.001*	2.16	1.72	2.71	<0.001*
Catastrophic injury or disease								
No	-	-	-	-	-	-	-	-
Yes	1.00	0.82	1.23	0.989	1.09	0.87	1.38	0.445
Relevant diseases								
Cancer	1.94	1.22	3.09	0.005*	1.59	0.95	2.67	0.080
Diabetes mellitus	1.63	1.33	2.00	<0.001*	1.41	1.14	1.74	0.001*
Level of severity of intellectual disability								
Mild	-	-	-	-	-	-	-	-
Moderate	0.70	0.60	0.82	<0.001*	0.73	0.62	0.86	0.000*
Severe	0.34	0.28	0.42	<0.001*	0.39	0.31	0.49	<0.001*
Very severe	0.33	0.26	0.42	<0.001*	0.42	0.32	0.54	<0.001*

^a^Level one: the most urbanized areas.

*p < 0.05.

Regarding age, the odds of Pap smear test usage tended to decline with increases in age. Pap smear test users aged ≥70 had an odds of use that was 0.36 times (95% CI = 0.19-0.69) that of the 30 to 39 year age group. Regarding the urbanization level, areas with Level 8 of urbanization (i.e., least urbanized) had a 1.52-fold (95% CI = 1.04-2.20) higher odds of use compared to regions with Level 1 of urbanization (i.e., most urbanized). Considering the economic conditions, using participants possessing premium-based monthly salary of < NT$15,840 as the reference group, the result show that people with NT$24,000 to NT$28,800 (OR = 1.79, 95% CI = 1.02-3.12) exhibited the highest likelihood to use. The odds of use among the aboriginal population was greater than that for the non-aboriginal population (OR = 1.73, 95% CI =1.08-2.76), and married users showed a 3.21-fold higher likelihood of use than that of unmarried users (95% CI = 2.59-3.97). DM patients had a greater odds of use compared to non-DM patients (OR = 1.41, 95% CI = 1.14-1.74). Using the “mildly disabled” group as a reference, the likelihood of use declined with the severity of disability (except for the group with extremely severe disabilities). The likelihood of use for the severely disabled group was 0.39 times lower than that of the mildly disabled group (95% CI = 0.31-0.49) (see Table 2).

## Discussion

To compare the Pap test use rate between women with intellectual disabilities and women without disabilities, we conducted further analysis. We matched women with intellectual disabilities with women without disabilities in the general population at a ratio of 1:5 by using age as the variable. Women with intellectual disabilities (N = 13,271, mean age 44.99 years ± 11.36) exhibited a significantly lower Pap test use rate (6.14%) than did (26.78%) women without disabilities (N = 66,355, mean age 45.15 ± 11.41).

The factors significantly associated with Pap smear test use identified in this study were similar to those identified in a previous study conducted by Huang et al. [16], who examined all disabled women aged over 30 years. In this study, which included women with intellectual disabilities, we observed that aboriginal women exhibited significantly higher Pap smear test use than did non-aboriginal women. This is because most aboriginal people live in rural areas, and the Pap smear test is provided through mobile medical care in rural areas. The mobile medical care program regularly travels through rural areas and government strongly encourages rural residents to use the free preventive medical services. We observed that physical disability was the most common type of disability (37.89%) [16]. Women with intellectual disabilities who underwent the Pap smear test had easier access to mobile medical care than did women with physical disabilities.

The results of this study conform to those of previous research, which contend that Pap smear test use reduces with increases in age [20-22]. Regarding residence, Pap smear test use rate among people residing in areas with lowest urbanized (i.e., Level 8 of urbanization) was significantly higher compared to that for people living in regions with most urbanized (i.e., Level 1). This finding was consistent with that reported by Chen et al. [23]. Furthermore; this phenomenon resulted from the implementation of NHI in 1995, before which studies showed that the use of Pap smear tests in urban regions exceed that in rural areas [24]. Lin et al. [25] further verified this point by comparing the Pap smear test usage of Taiwanese women before and after the implementation of NHI in 1995. In addition, Taiwan implemented the Integrative Delivery System (IDS) program in 1999. The primary function of the IDS program was to improve the medical accessibility of rural areas, thereby enhancing the health care acquisition of residents in these areas. The cervical cancer screening rate was among the primary criteria for assessing project effectiveness [26], thereby increasing the use of Pap smear tests in rural areas [21,23,25].

Regarding economic aspects, the Pap smear test usage rate among women with intellectual disabilities rose with increases in their premium-based monthly salary, exhibiting a positive correlation. The usage rate for people with a premium-based salary of > NT$15,840 (including the dependent population) was higher than that for users with monthly salary of < NT$15,840. This result was consistent with that reported in a U.S. study (2010), which contended that disabled persons with high incomes exhibit greater Pap smear test usage [27]. The usage rate among the aboriginal population exceeded that of the non- aboriginal population (OR = 1.73, 95% CI =1.08-2.76). Despite the overall middle-to-low income of the aboriginal population (which may reduce utilization rate), their Pap smear test usage rate was greater compared to the non- aboriginal population because the Taiwanese government has made considerable efforts to reduce the gaps in income and health care between urbanized and rural areas. A great proportion of aboriginal people reside in rural areas, and the medical vehicles that provide Pap smear tests regularly visit these areas to offer health care services, thereby improving the Pap smear test usage rate among aboriginal women. The Pap smear test usage rate of the married population was substantially greater than that of the unmarried population, which is consistent with the findings reported in previous studies [6,22,27]. This phenomenon may be because of the common misconception held by the public and physicians that unmarried people do not or seldom engage in sexual activity and, thus, are less susceptible to cervical cancer. In addition, Asian women generally possess more conservative concepts compared to Western women, and tend to believe in maintaining virginity until marriage [28]. Consequently, women in Asia are less likely to accept this type of preventative health care before being sexually active. Thus, the Pap smear test usage rate for the unmarried population is substantially lower than that for the married population.

Persons with DM exhibited a higher odds of Pap smear test use compared to that for people without DM (OR = 1.41, 95% CI = 1.14-1.74). Pandey et al. [29] found that DM increases cancer risks. Specifically, type 1 DM directly affects cervical cancer. Whether DM patients are more likely to receive Pap smear tests for this reason requires further investigation. In addition, DM patients must undergo regular health checks; therefore, they have more contact with paramedics and are more likely to receive Pap smear tests following the advice or information provided by paramedics. Regarding the severity of disability, the usage rate of mildly disabled people was substantial, which conforms to the findings of a study conducted in 2010 on women with intellectual disabilities [30]. For primary care givers or paramedics, communication difficulties may occur when administering Pap smear tests to women with severe intellectual disabilities. Furthermore, substantial time and manpower are required for this process; thus, the provision of preventative health care to severely disabled women is extremely difficult.

We provide the following recommendations: (a) The knowledge of the Pap smear test of women with intellectual disabilities who exhibit a low usage rate and that of these women’s primary care givers must be enhanced. Insufficient relevant knowledge is a factor associated with women neglecting to undergo Pap smear tests [31]. McAvoy and Raza [32] indicated that individual interviews and family viewing of educational videos are the most effective methods for enhancing knowledge of Asian women regarding Pap smear test. (b) Rewards proportional to the disability severity of patients should be provided to physicians as an incentive increasing physicians’ willingness to assist women with intellectual disabilities in undergoing Pap smear tests. Lennox et al. [15] stated that providing health care to disabled people involves considerable difficulties (e.g., communication difficulties, poor compliance, examination difficulties, and excessive time consumption); therefore, payment increases may be a practical and effective solution for enhancing the quality of health care provided by physicians. (c) Female physicians should be encouraged to receive service training to provide health care to specific patient groups, thereby increasing the opportunities of women with intellectual disabilities to receive Pap smear tests administered by female physicians. Fylan [31] determined that women do not undergo Pap smear tests because of concerns regarding awkwardness, pain, and fear. A previous study indicated that, overall, women prefer to undergo Pap smear tests administered by a female physician and that being administered the test by a female physician increases their willingness to undergo Pap smear tests [33].

### Limitations

This study referenced numerous databases to investigate the Pap smear test usage of women with intellectual disabilities based on data from 2008. However, the referenced databases did not include information regarding the participants’ health beliefs and lifestyles. Therefore, this study is limited by the difficulty of further analysis. In addition, data obtained from the NHI Research Database has inherent limitations; specifically, the premium-based monthly salary does not completely reflect the real salaries or income of the women with disabilities.

## Conclusion

This study provides significant baseline information regarding the Pap smear test usage of women with intellectual disabilities, and the relevant influencing factors. The results of this study indicated that in 2008, women with intellectual disabilities who were aged 30 years and above exhibited a 4.83% Pap smear test screening rate, significantly lower than that for the general population of women. Thus, policy intervention is required to further improve the health inequities of women with intellectual disabilities. Factors including an older age, urbanized residence, low income, unmarried marital status, absence of DM, and severe disability were associated with a lower probability of Pap smear test use.

## Abbreviations

CI: Confidence interval; DM: Diabetes mellitus; IDS: Integrative delivery system; IQ: Intelligence quotient; NHI: National health insurance; NT$: New Taiwan dollar; OR: Odds ratio; SAS: Statistics analysis system; SIR: Standardized incidence ratio; U.K.: United Kingdom of Great Britain; U.S.: United States.

## Competing interests

The authors declare that they have no competing interests.

## Authors’ contributions

WCT and PTK conducted the study design. SMY drafted the manuscript. PTK conducted the statistical analysis. WCT was the supervisor of the study and revised the manuscript critically for important intellectual content. All authors read and approved the manuscript.

## Pre-publication history

The pre-publication history for this paper can be accessed here:

http://www.biomedcentral.com/1472-6963/14/240/prepub
